# Rotating carbon nanotube membrane filter for water desalination

**DOI:** 10.1038/srep26183

**Published:** 2016-05-18

**Authors:** Qingsong Tu, Qiang Yang, Hualin Wang, Shaofan Li

**Affiliations:** 1 Department of Civil and Environmental Engineering, University of California, Berkeley, CA, USA; 2 State Environmental Protection Key Laboratory for Environmental Risk Assessment and Control on Chemical Process, East China University of Science and Technology, Shanghai, China

## Abstract

We have designed a porous nanofluidic desalination device, a rotating carbon nanotube membrane filter (RCNT-MF), for the reverse osmosis desalination that can turn salt water into fresh water. The concept as well as design strategy of RCNT-MF is modeled, and demonstrated by using molecular dynamics simulation. It has been shown that the RCNT-MF device may significantly improve desalination efficiency by combining the centrifugal force propelled reverse osmosis process and the porous CNT-based fine scale selective separation technology.

In recent years, impressive breakthroughs have been made towards using nanoscale structural materials, such as carbon nanotubes (CNTs)^1^,^2^,^3^,^4^,^5^,^6^,^7^ and porous graphene membranes^8^,^9^,^10^,^11^,^12^,^13^,^14^, in water purification and desalination applications. One of the main advantages for such approach is that the water permeability through CNT channel can be three orders of magnitude higher than that of conventional membrane devices^15^,^16^.

In 2001, Hummer *et al.*^17^ first used molecular dynamics (MD) simulation to demonstrate that water molecules can pass through the hydrophobic CNT channel of 8.1(Å) diameter. Since then, the nanoscale hydrodynamics behaviors of CNT-based devices have been extensively studied. Both experimental studies^15^,^18^,^19^ as well as MD simulations^20^ have indicated that the water permeability inside CNTs or through graphene can be 4~5 orders of magnitude higher than that in conventional membrane devices, which is often based on continuum hydrodynamic theory. In 2008, Fornasiero *et al.*^6^ studied the ion selectivity of CNTs, and they showed that ion rejection can be controlled by diameter of CNT channel, which functions like a bio-channel^21^. Recent studies have revealed that ion hydration is the primary reason for CNT selectivity, and this is because that the average sizes of *Na*^+^ and *Cl*^−^ ions inside the CNT are about 4.85 and 7.17 coordinate number respectively^22^, which is about 10~12 Å in diameter^23^,^24^. On the other hand, the water molecular only has a size of 2.8 Å in diameter inside the CNT. Thus, the combination of the high permeability of water molecules in CNTs and the large difference in hydrated sizes between water molecule and ions make the CNT or graphene an excellent structure material for water-ion separation. Indeed, much progress has been made in this field in the past decades, and the CNTs based membrane technology has been hailed as a paradigm shift in advance of desalination and water purification technology^1^,^25^,^26^,^27^.

However, in the existing CNT-based desalination technology, high energy input is needed to provide high hydrostatic pressure^27^, in order to drive water molecules through the sub nanometer-diameter CNT channel. Moreover, in these approaches, ions are separated from fresh water molecules at the entrance of the CNT channel, which will cause serious fouling problem as solute accumulating at the entrance and finally block the entrance^28^,^29^.

In parallel with the developments of CNT-graphane membrane technology, in past few years, some new physical phenomena about CNTs have also been discovered, and they have been used to develop CNT-based microfluidic devices. For instance, Bailey *et al.*^30^,^31^ discovered that when applying voltage at ends of a CNT, it will start to rotate because of the electron wind effect. Subsequently, several groups^32^,^33^,^34^ have shown the feasibility and strategy to build a charge-driven CNT water pump by applying electric field to CNT. It has also been reported that under such condition CNTs can reach to a very fast angular velocity at around 200–300 rad/ns^35^. This will produce an enormous centrifugal acceleration on the CNT wall almost up to 10^11^ *g*, and this value surpasses by five orders of magnitude acceleration in the fastest centrifuges available today^36^. Additionally, Feng *et al.*^37^ studied a rotating chiral type CNT under electric field, and they observed a rotating-induced axial pressure in the CNT, which can drive water molecules flowing through CNT without a dependence of high external pressure.

In this work, by utilizing the physical features of the rotating CNT and the vacancy modified CNT, we propose a novel nanoscale fluidic device — a rotating carbon nanotube membrane filter (RCNT-MF) — to resolve some of the critical issues in CNT-based membrane technology. The design concept of the proposed RCNT-MF nanofluidic device is illustrated in Fig. 1. There is a CNT motor at the top of the proposed desalination device, which provides power to keep the CNT filter rotating. The functional part of RCNT-MF is a partial double-wall CNT system, and when electric voltage is applied to the outer CNT it may exert a torque to the inner CNT. This may be done by properly choosing chiral vectors for the outer and the inner CNTs so that the outer wall may conduct electricity while the inner wall cannot. For the desalination purpose, we make many small holes with the diameter less than 1 nm on the wall of the inner CNT, which can be done by using the ion bombardment method^38^ or the e-beam lithography^39^. For the chiral type CNTs, the rotating motion will generate a negative pressure that draws the salt water into the top entrance of the RCNT-MF; once salt water enters into the CNT membrane channel, the centrifugal force generated by CNT rotation will throw the fresh water molecules out from the pores on the CNT wall, while keeping NaCl ions inside the rotating CNT pipeline if the pore size on the CNT wall is carefully selected. Since the chiral type CNT can generate a pressure gradient along the axial direction of the rotating CNT^37^, the remaining salt water inside CNT will be pushed out naturally from the bottom of the device. For practical purpose, one may want to make the diameter of CNTs very large, say up to 1000 nm or even larger, and from this perspective the CNT wall may be viewed as a curved graphene membrane structure or graphene shell structure. The biggest advantage of the proposed rotating CNT system is that every pore serves as a water transportation channel, and they can perform desalination independently and in parallel; meanwhile the number of pores on the wall increases as the length of CNT increases, as long as the system is structurally stable. Depending on the length of the CNT and stable porosity value of the CNT, one can open more than thousands of pores in a giant CNT membrane shell, which can fully take advantage of the high length-diameter ratio of CNT material and increase desalination efficiency significantly.

From the perspective of practical applications, we would like to comment the scale-up feasibility of the proposed device. Even though the present work is only a computational modeling and simulation of the working principle of the proposed desalination device, the scale-up or macroscale manufacturability of such devices is highly possible. First, the state-of-the-art nanotechnology has shown promising potential to solve some key issues for the scale-up CNT production, For instance, the Arc/Laser method^40^ and chemical vapor deposition (CVD) method^41^ have already been able to produce large scale CNTs; and there have been reported that Ion bombardment method^38^ and e-beam lithography method^39^ can make pores with diameter less than 1 nm on CNT walls. Recent fabrication techniques have enabled the arrangement of aligned tubes of controllable sizes incorporated across a polymer film to form a well-ordered nanoporous membrane structure^42^,^43^. Second, the nanoscale motor-driven rotating CNT design is not essential for the desalination strategy that is proposed in this work. What is essential to the nanoscale separation process is the combination of the centrifugal force field and the selectivity of nanoscale pores on the graphene.

In fact, we can design macroscale graphene filters that work under the same physical principle. For example, we can fabricate a macroscale pipe with many tiny holes on its surface, and then paste porous graphene membrane patches to cover those surface holes on the macroscale pipe. By rotating the pipe, the water molecules inside the pipe may permeate through the porous graphene membrane separating themselves from salt ions. In fact, the macroscale pipe pasted with porous graphene membrane patch may serve as the guideline design for fabricating a macroscale (scale-up) rotating filter proposed in this work. The nanoscale desalination model presented in this paper is only for illustrating centrifugal desalination concept in the convenience of computational resource, because using molecular dynamics to simulate a macroscale object or process is still a formidable task today.

In this study, we use the MD simulation to investigate desalination performance of a rotating CNT desalination device. We first investigated the transversal permeation properties of water and ions as a function of pore size and angular velocities. Then by selecting an optimized pore size and angular velocity, we study the effect of pore numbers on the permeation rate. Last, we examined the desalination efficiency and performance of the proposed RCNT-MF nanofluidic device and compared the overall efficiency of this model with other methods.

## Methods: Modeling and Simulation

**Figure 2:**
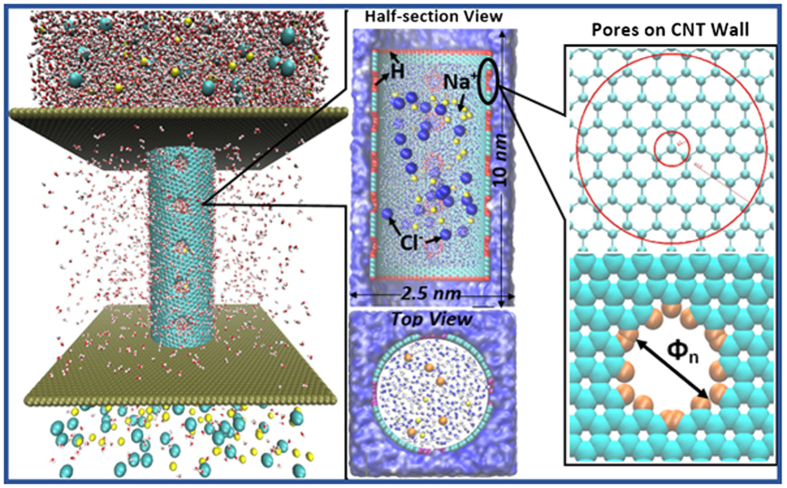
Configuration and working principle of RCNT-MF device (*Left*); the half-section view and top view of the Carbon Nanotube (*Middle*); the description of pores onthe CNT wall (*Right*).

In this study, we are focusing on the following critical factors: (1) the dependence of clean water permeation rate on the diameter of pores on the CNT wall, (2) the angular velocity of the CNT, and (3) the number of pores on the CNT wall. As shown in Table 1 and Fig. 2, we build 11 CNT simulation models with a chiral vector (16, 32) (about 3.315 nm in diameter) and 10 *nm* in length to simulate their desalination performance. Five different diameters of pores (**Φ**_*n*_) on CNT wall are chosen to study the pore size effect; five different angular velocities are applied on CNTs to study the effet of rotation speed, and three different pore numbers are selected to study the pore number effect. Pores with different radius are created, ranging from 0.28 nm to 0.57 nm as shown in Fig. 2d, with commonly used hydrogen atoms decorated at the dangling carbon atoms both on the hole and the CNT.

We perform all-atom molecular dynamics simulations to the proposed desalination device. The entire desalination structure is solvated in water environment, which contains 13800 water molecules (13800 oxygen atoms and 27600 hydrogen atoms). Initially (t = 0), there are 4900 water molecules outside the CNT, and the salt water inside the CNT consists of total 4900 pure water molecules, 16 Na+ ions and 16 Cl− ions, which is comparable to the seawater salinity 35 g/L. The diameter of the model CNT is set at 3.315 nm, which consists of 2500 Carbon atoms, and it is large enough to allow salt water (*H*_2_*O* molecules and Na+ Cl− ions) flowing in and out of the CNT device freely like in bulk environment, even though in application one may build a much larger RCNT-MF device.

Pores with different diameters are created, ranging from 0.56 nm to 1.14 nm as indicated in Table 1. The dangling bond or dangling carbon atoms at the edge of the pore are decorated with hydrogen atoms.

We have used the open source molecular dynamics simulation software GROMACS^44^ in the MD simulation. The OPLS force field^45^ is adopted to describe the bond strength between different atoms and molecules, which are characterized by the following atomistic potential,


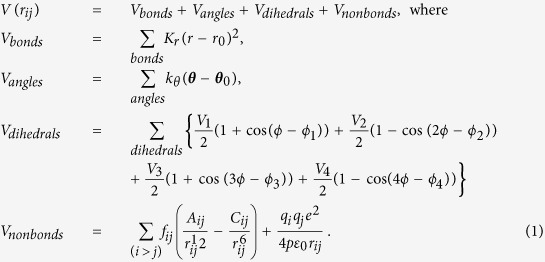


The meanings and choices of all parameters in above equations can be found in ref. 45. In simulations, we have used both SPC/E water molecule model as well as TIP3P water molecule model^46^. Differences in simulation results between the two are small, while the computation used the SPC/E rigid water molecule model is much faster than that used TIP3P model, because in SPC/E model only the non-bonded molecular interaction is considered. The CA type is used for carbon atoms in the CNT model. The size of the simulation box depends on the dimensions of the CNT, and periodic boundary conditions in all directions are enforced. The particle mesh Ewald method^47^ is used for calculating electrostatic interactions, and the cut-off distance for the Lennard-Jones (LJ) interactions is set at 0.8 nm. The MD simulation is performed in three steps: The MD system is first relaxed to minimize its total free energy for 50 ps. After that we follow an NVT ensemble simulation for another 50 ps by using the Nose-Hoover thermostat^48^ to achieve an equilibrium temperature at 300 K. Once the MD system is in the desired equilibrium state, we then start the desalination simulation for another 8 to 10 ns. The MD system usually becomes stable after about 1 ns, and we only use the last 5 ns of the trajectories for equilibrium data analysis. The integration time step is chosen as 1 fs, and the data are collected every 0.5 ps. The neighbor list is updated every time step to avoid intrinsic errors^49^. The porous CNT system is subjected to a prescribed angular velocity ranging from 1.75 rad/ns to 175 rad/ns in the simulation. The selection of angular velocity is based on the fact that a critical rotation speed is needed to generate sufficient centrifugal pressure to overcome osmotic pressure to separate pure water molecules from the salt solvent. A detailed thermodynamic analysis will be presented in a separated paper on how to choose such critical angular speed. The CNT may be assumed to be stationary in a rotating frame, and the rotational motions of water molecules, Na+, and Cl− ions are generated by the inter-molecular interaction between the CNT with water molecules and with ions, and the complex interactions among themselves. We analyze the statistical data sampled during the 1 ns to 10 ns range in order to obtain meaningful converged results for the time scale involved in the MD calculations.

## Results

The basic desalination mechanism of RCNT-MF is that the centrifugal force will counter-balance the osmotic force to realize the reverse osmosis process. It is true that ions are also subjected the centrifugal force, which is along the radial direction, and it will tend to push ions out of CNT as well, but the size of hole is small enough to keep them inside. On the other hand, there will be a Coriolis force in the tangential direction, which will push away any bigger molecule clusters blocking the pores. This is the reason why we conclude that the rotating action induced Coriolis force may have acted as an anti-fouling agent, as what have been observed in the MD simulation. In the MD simulations, we have not observed any fouling problems inside the RCNT-MF. We think that the big ion clusters staying in the front of pores may be swept away by the Coriolis force, which may be another advantages of the proposed nano-fluidic desalination device.

**Figure 3:**
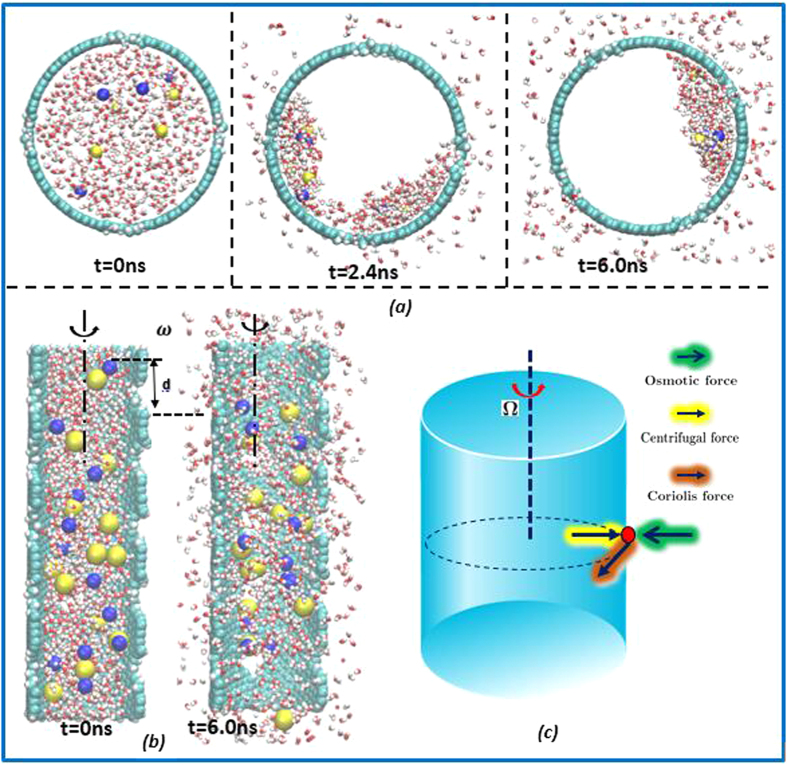
Time sequences from MD simulation and snapshots are taken from top view (left) and half-section side views (right) for the CNT (16, 32) with length 11 nm, 20 pores on the graphene wall and angular velocity ***ω*** = 174.5 *rad*/*ns*. (**a**) Perspective in cross-section view; (**b**) Perspective in longitudinal view, and (**c**) Schematic illustration of force diagram with the directions of the Centrifugal force, the Coriolis force, and the Osmotic pressure. Note that water molecules that are initially outside the CNT membrane are hided.

**Figure 4:**
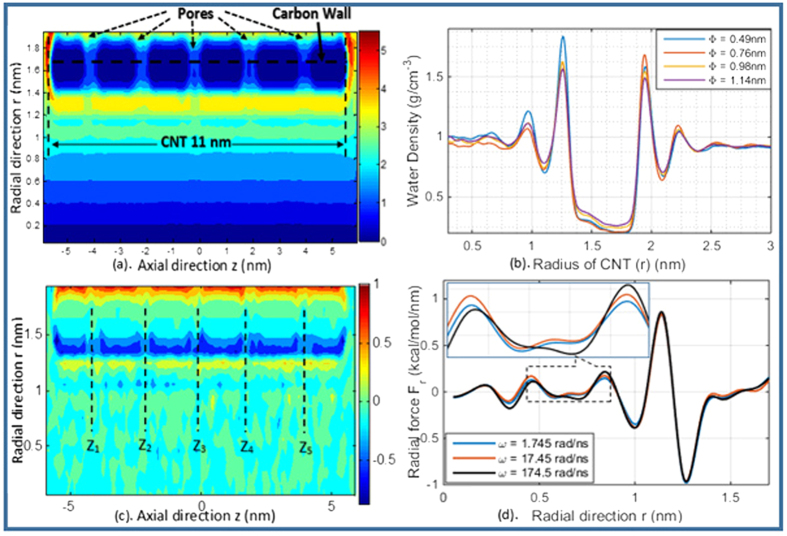
(**a**) Water molecule density profile of Model 3 (pore size **Φ** = 0.76 nm, angular velocity ***ω*** = 174.5 rad/ns); (**b**) Water densities of Model 1–5 (**Φ** is different, but angular velocities are ***ω*** = 174.5 rad/ns) along radial direction; (**c**) Average radial force profile of Model 3 (pore size **Φ** = 0.76 nm, angular velocity ***ω*** = 174.5 rad/ns); (**d**) Radial force distributions in MD Model 6 to 9 (**Φ** is fixed to be 0.76 nm, but angular velocities are different) along radial direction.

Figure 3 shows a sequence of snapshots of a MD simulation of salt water flowing in a rotating (16, 32) nanotube. One may observe that: (1) Water molecules inside a rotating CNT are more likely to accumulate close to the graphene walls; (2) The centrifugal force will drive clean water molecules flowing outward through the pores on the graphene wall of the rotating CNT membrane; (3) NaCl ions cannot pass through the pore on graphene wall, because the size of the pore is chosen to prevent them passing through the holes, even though they may move towards the graphene wall, and (4) A more detailed inspection shows that ions are moving longitudinally as well, with a velocity of 1.36 nm per 6 ns (0.227 m/s), and the moving direction is determined by the chiral direction of the CNT. We sketched the other external forces acting on in Fig. 3(c) besides the molecular interaction force. It may be note that the osmotic force is a thermodynamic force, whereas the centrifugal force and the Coriolis force are Newtonian force. The centrifugal force acting on the particle *i* on the fluid filed may be estimated as


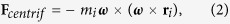


where ***ω*** is the rotating angular velocity. However, this is a simplified picture, and in actual flow field the centrifugal force effect on individual molecule depends on the complex molecular interactions. In Fig. 4(b–d), we show the actual average radial force field inside the CNT. If we assume that the angular velocity of the rotating frame is along z-axis, each water molecule will have relative radial velocity, and there will be an effective Coriolis force acting on each water molecule, which may be estimated as,


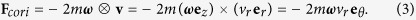


It is noted that just like the centrifugal force effect, the Coriolis force effect on each molecule also comes from the interaction among different atoms and molecules. On the other hand, it acts on the tangential direction of the CNT wall (see Fig. 3(c)). Thus we speculated that the Coriolis force may play an important rule as an anti-fouling agent. A detailed and quantitative numerical analysis of the Coriolis force effect will be reported in a separated paper, because the Coriolis force may be convoluted with the relative water molecule velocity in the chiral direction on the CNT wall, which is apparently a complex issue.

### Water density profile

We have examined the water density profiles in both axial and radial directions, and plotted them in Fig. 4(a,b). The water density profile shown in Fig. 4(a) is obtained from a typical run of the rotating nanotube with pore size **Φ** = 0.76 *nm* on the CNT wall and the angular velocity ***ω*** = 174.5 *rad*/*ns*. Figure 4(b) shows the water molecule distribution along radial direction under different pore diameters. One may find that water density keeps on increasing as *r* increasing from 0.0 to 1.4 nm because of the centrifugal force. However, because of the van der Waals interaction, the CNT wall will also repel water molecules when they come too close to the wall, and a well-developed boundary layer is formed close to the wall. The only passage that enables water molecules passing through the rotating CNT is the opening pore. Figure 4(b) also shows that water density profile shifts outward as ***ω*** increases, because high angular velocity ***ω*** will generate larger centrifugal force on water molecules, which will overcome and exceed the van der Waals repel force from the CNT wall.

### Dynamics of the system

The time average radial force acting on water molecules between *r*_0_ < *r* < *r*_1_ along radial direction is calculated by using the following formula,


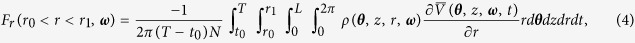


where L is the length of the CNT; 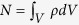; *ρ*(***θ***, *z*, *r*, ***ω***) is the water molecule number density i.e. *N*/*V*, which can be obtained from the water density profile in the simulation; *t*_0_ is the time duration of relaxation, and T is the total time of simulation. The atomistic potential in the rotating frame is defined as


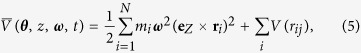


where *m*_*i*_ is the mass of the i-th atom, ***r***_*i*_ is its position vector, ***ω*** is the prescribed angular velocity, **e**_Z_ is the unit vector in Z-axis, and the scalar *r* and *z* represent the radial coordinate and axial coordinate; and 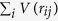 is the standard OPLS potential as described in Equation (1).

The average force profile is shown in Fig. 4(c). Examining the radial force at the selected points: *z* = [*z*_1_, *z*_2_, *z*_3_, *z*_4_, *z*_5_] (locations corresponding to pore sites on CNT wall) and averaging all the five values, we find that the average radial force is a function of CNT radius and angular velocity as shown in Fig. 4(d). The radial force increases as ***ω*** increases if *r* < 1.2 *nm*, and it stays a constant value if *r* > 1.2 *nm*. This is because that the centrifugal force dominates in the first region, and therefore it is a function of ***ω***. On the other hand, the van der Waal force dominates in the second region, and it is characterized by the interaction between the CNT wall and water molecules. We can calculate the time for a single *H*_2_*O* molecule passing through a pore on the graphene wall by using the formula,


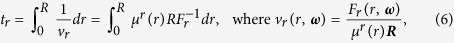


where *μ*^*r*^(*r*) is the friction coefficient of the CNT per unit length along radial direction. Because of the rotating motion of the device and the resistance force from nanopores, the radial velocity is not constant. Therefore, the equivalent viscosity in the radial direction will change as a nonlinear function of radius *r*. However, if we assume this value has the same magnitude as the viscosity along axial direction, this is the case that has been well studied and quantified by Feng *et al.*^37^ e.g. for the case of a (16, 32) chiral type CNT with *μ* = 7.0 × 10^−4^ *kgs*^−1^ *m*^−1^, and we can obtain the time needed for transverse water diffusion, which is about 100 times smaller than the time needed for longitude motion.

We also calculated the time average axial force and the time duration *t*_*z*_ for a flux of salt water molecules passing through the rotating CNT by using the following formulas,


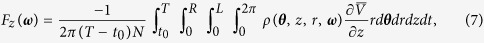



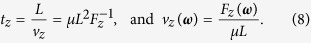


The axial motion is the same as what described in^37^. Therefore, we used the same axial viscosity in the above equation, i.e. *μ* = 7.0 × 10^−4^ *kgs*^−1^ *m*^−1^. The computed results are tabulated in the third and the fourth columns of Table 2. It may be noted that this force should be zero if there is no rotation, because in that case no external force is applied at axial direction. After a chiral type of CNT starts to rotate, axial force will automatically appear because of unsymmetrical lattice structure of the chiral CNTs, and this force is a function of angular velocity ***ω*** with a local direction that is the same as the direction of chirality of CNT. In other words, the axial force is the projection of the chiral direction force that is generated by the rotating chiral CNT.

Finally, the fresh water flux rate of one single pore along radial direction is a function of pore size **Φ** and angular velocity ***ω***,


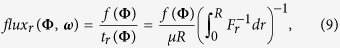


where *f*(**Φ**) is a function to describe the dependency of flux on **Φ**.

If more pores are created on the CNT wall, say *N*_*p*_, the total number of cumulative fresh water molecules flowing out CNT from all pores during the time when the same amount salt water passing through the CNT filter becomes,


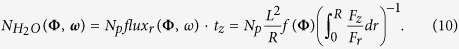


To obtain the highest possible water permeability through the CNT membrane filter, we may seek the optimal value for both **Φ** and ***ω***.

### Effects of pore size on water flow rate

**Figure 5:**
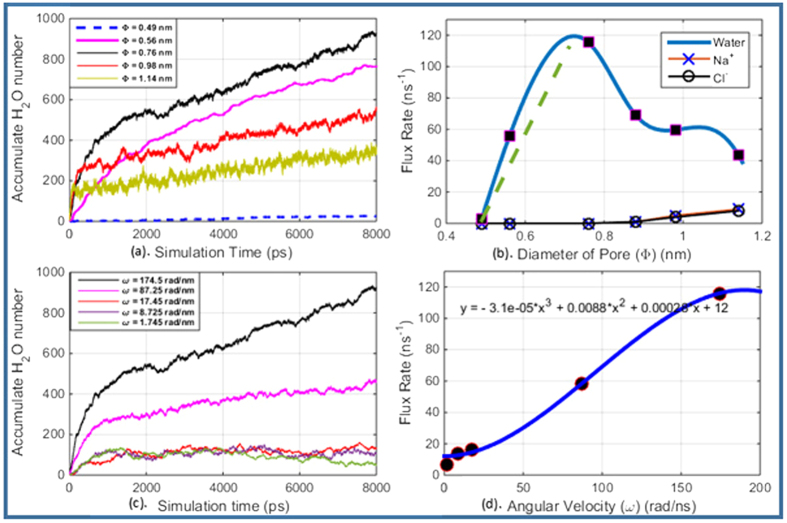
(**a**) Accumulated water molecules in Model 1 to 5 during the simulation time; (**b**) Average molecular flow rates for both water and ions in Model 1 to 5; (**c**) Accumulate water molecules in MD Model 6 to 9 during the simulation time, and (**d**) Average water flow rate in simulation Model 6 to 9.

From MD simulation results, we find that the cumulative water flux for each permeable pore on graphene wall of the RCNT-MF under a given angular velocity (174.5 *rad*/*ns*) is a linear function of time as shown in Fig. 5(a). The result shows that water flux is highly dependent on the size of permeable pores on the CNT wall; however, we have found that the water flux first increases as the pore size increases until a critical pore diameter, D = 0.76 nm, is reached, then it starts to decrease even if we increase the pore diameter as shown in Fig. 5(b). By checking the movement of ions for the cases of different pore sizes, we found that the pore starts to be blocked by ions at the critical diameter *D* = 0.76 *nm*, but ions still remain inside the CNT until the pore diameter reaches to *D* = 0.88 *nm*. As ions block or flow through the pores on the CNT wall, some “water channels” are either blocked by ions or become “ion channels”, which means that less water molecules can pass through those channels. Thus, as the pore size exceeds the critical value the number of “water channels” decreases, therefore water flux rate will decrease. In other words, RCNT-MF has a perfect salt rejection property before the pore size reaches to the critical value. On the other hand, the pore diameter should not be smaller than 0.5 nm to ensure water molecules can pass thought the pores on the CNT wall. It may be noted that this value is smaller than the results reported by Hummer *et al.* (0.8 nm)^17^ for a water molecule entering a single wall CNT longitudinally. The pore diameter should not be bigger than 0.88 nm to ensure Na+ and Cl− ions cannot flow out of the CNT, which is also much smaller than the results reported by Feng *et al.*^37^, and Beu *et al.*^50^, in which they reported that the maximum diameter of a single wall CNT to block Na+ and Cl− ions entering the the CNT in longitudinal direction is 1.2 nm. Comparing with the previous results in the literature, we find that first the pores created on the CNT wall have the similar water diffusion function as that of the longitudinal CNT channel reported in literature. Second, because of the centrifugal force effect, the critical radii for both water molecules and salt ions flowing out the CNT desalination device are smaller than that of the longitudinal CNT channel approach.

We recorded the fresh water flux rate for each pore as a function of pore diameter. The flux rate *flux*_*r*_ is “switched on” once the pore size exceeds the minimum diameter **Φ** = 0.49 *nm*. Above this value, the flux rate is proportional to the pore diameter **Φ** until it reaches to the critical diameter **Φ** = 0.76 *nm*. We obtained the following expression by fitting the numerical results for **Φ** from 0.49 nm to 0.76 nm,


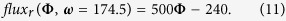


As shown in Fig. 5(b), this formula only works for those pores whose diameter is smaller than 0.76 nm, and it applies to those cases that the filtering pores are large enough to allow at least one column of water molecules passing through. The linear relation between water flux and pore diameter suggests that in these cases the pore size determines the desalination efficiency for a given rotating speed.

### Effect of angular velocity on water flow rate

To study the effect of angular velocity on water flow rate, We investigated fresh water flux rate versus CNT angular rotating velocity when the pore size is fixed. In this case, the clean water flux rate is also a function of angular velocity. For the case of a fixed pore diameter **Φ** = 0.76 *nm*, we extrapolated the relation between the clean water flux and the rotating frequency by using MD simulation results. The simulation results are displayed in Fig. 5(c,d).

Figure 5(d) displays the relationship between the clean water flow rate versus the value of ***ω*** before it reaches the optimal value. It shows that the water flow rate has a cubic relation with ***ω***; we can directly obtain the following expression by converting the data shown in Fig. 5(d),


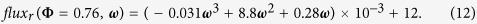


Combining Equations 11 and 12, we can obtain the unified expression for clean water flow rate as a function of **Φ** and ***ω***:


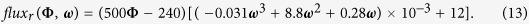


This relation is valid for the cases that the pore diameter is between 0.49 *nm* to 0.76 *nm*.

### Effects of number of pores on water flow rate

**Figure 6:**
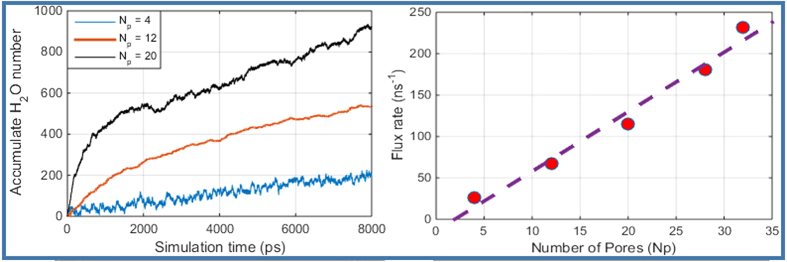
(**a**) Accumulated water molecules in Model 3, 10 and 11 during the simulation time, and (**b**) Average water flow rate of as a function of number of pores.

The water permeability under different pore numbers are studied. For the pore diameter **Φ** = 0.78 *nm* and the angular velocity ***ω*** = 174.5 *rad*/*ns*, we plot the results in Fig. 6(a,b). From Fig. 6(a), one may find that the number of output fresh water molecules increases linearly with the simulating time. Figure 6(b) shows fresh water flux rate also increases linearly with number of pores. These results show that each pore on CNT wall performs independently and contributes equivalent water flux to the whole permeability of the system. In principle, for a given salt water in-flow rate, the output fresh water flow rate may approach to the same order of magnitude if the in-flow ratel, if a sufficient number of pores are created on the CNT wall, which implies that the overhead of the desalination process can be made very small.

### Desalination efficiency

To study the overall desalination efficiency, we have calculated energy change in the MD system at angular velocity ***ω*** = 45 *rad*/*ns*. We have found that the potential energy of the system does not change much, while the kinetic energy of the system change significantly. To estimate the energy consumption, we have calculated the kinetic energy increase of each water molecule at the time it is moving out the CNT. After the first 10 ns, the total number of water molecules coming out of the CNT is 267, and the total kinetic energy of all water molecules moving out in the first 10 ns is: *K* = 4.012 × 10^−18^ *J*, while the reference kinetic energy at the zero angular velocity (of CNT) with other conditions remaining the same is: *K*_*o*_ = 3.974 × 10^−18^ *J*.

Thus the energy increase is: Δ*K* = *K* − *K*_*o*_ = 3.8 × 10^−20^ *J*. Therefore, the energy consumption efficiency of the desalination is


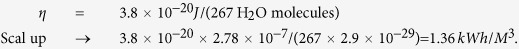


This is a very promising result in reverse-osmosis desalination process, even thought this result may slightly underestimate the energy consumption, because that it does not include the overhead energy consumed in the system. To estimate the overhead energy including the industrial process overhead is out the scope of this paper. This issue will be thoroughly investigated in another work that will be reported in a separated paper.

Besides the advantages of low energy consumption and high salt rejection, what makes the RCNT-MF model very attractive is its superior ability of flow rate enhancement. In the model presented in this paper, there are only 20 pores along an 11 nm long CNT; however, in the real manufacturing process, the nanotube membrane structure can be several micron long, e.g.^51^, and the radius of the rotating CNT may be up to 100 nm or even larger. With such long and large CNT membrane, thousands of pores can be created on their graphene walls, which, in principle, can make the water desalinating efficiency several orders of magnitude higher than the CNT desalination devices reported in the literature. According to Equation (7), theoretically, this efficiency enhancement may increase without limit, because the attractive force between salt ions and water molecules will increase as the salinity of the salt water increases as indicated in Equation (8).

## Conclusions

In summary, the MD simulation results presented in this paper show that the proposed RCNT-MF device provides a distinctly significant tool in reverse osmosis desalination comparing to the current CNT desalination device^50^. In addition to provide a viable alternative to the external pressure driven graphene membrane desalination devices, the proposed model has the ability of anti-defouling, because that the rotating system provides a Coriolis force that is acting at the tangential or the circumferential direction of the CNT wall, which will push off ions that are stuck near the site of nanoscale pores. In particular, the simulation results show that the salt water can continuously flow through RCNT-MF without additional external pressure because of the self-propel mechanism of the chiral type CNTs, which ensures a continuous desalination process. Second, because RCNT-MF uses large diameter CNTs as the key component of the desalination device, it takes advantages of both CNT and the graphene membrane, which will greatly increase the desalination efficiency. Considering all these factors, the proposed Rotational Carbon Nanotube Membrane Filter desalination model may provide a promising nanofluidic device for salt water desalination, purification, and separation.

Besides the application to the reverse Osmosis separation, the potential application of the proposed nanoscale separation model is multitude. The proposed RCNT-MF can be applied to other areas by changing the pore size on CNT wall or changing the angular velocity to create different types of nano-channels for different purposes, such as as separation of isotopes of Uranium, separation of virus from blood cells, and separation of much bigger organic molecules, e.g. water/benzene, methanol/benzene, ethanol/benzene, acetone/benzene mixtures, and inorganic heavy metals from wastewater simply by adjusting the diameter of pores on CNT wall.

## Additional Information

**How to cite this article**: Tu, Q. *et al.* Rotating carbon nanotube membrane filter for water desalination. *Sci. Rep.* **6**, 26183; doi: 10.1038/srep26183 (2016).

## Figures and Tables

**Figure 1:**
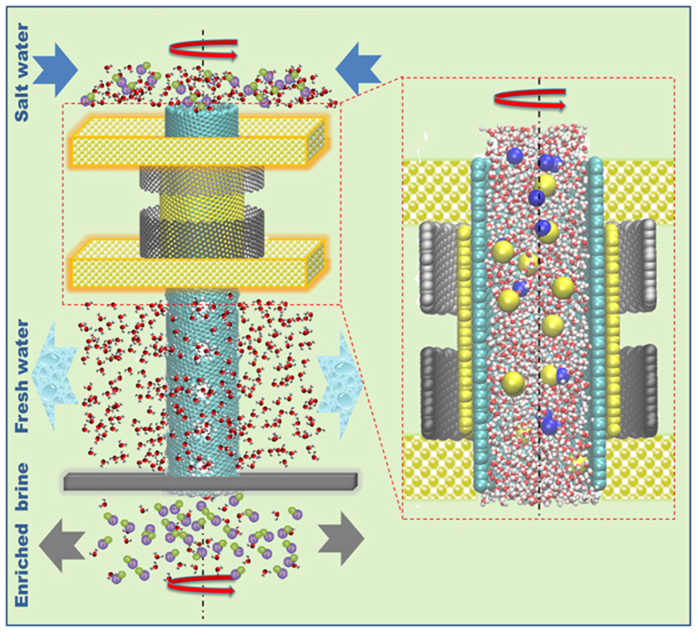
Illustration of the design concept of the rotating CNT membrane filter and its desalination mechanism based on the working principle of the double wall rotating CNT^52^.

**Table 1:** Description of all MD models.

Model Name	Pore Diameter (nm)	Angular Velocity (rad/ns)	Pore number
Model 1	0.49	174.5	20
Model 2	0.56		
Model 3	0.76		
Model 4	0.98		
Model 5	1.14		
Model 6	0.76	87.25	20
Model 7		17.45	
Model 8		8.725	
Model 9		1.745	
Model 10	0.76	174.5	4
Model 11			12

**Table 2:** The average axial force and velocity under different angular velocities.

Angular velocity *ω ns*^−1^	Axial force *F*_*z*_ pN	Axial velocity *v*_*z*_ nm/ns	*t*_*z*_ ps
0.175	43.56	5.66	1944
1.75	44.12	5.73	1920
17.5	44.61	5.79	1899
